# Extrusion Cooking Effect on Carbohydrate Fraction in Novel Gluten-Free Flours Based on Chickpea and Rice

**DOI:** 10.3390/molecules27031143

**Published:** 2022-02-08

**Authors:** María Ciudad-Mulero, Erika N. Vega, Patricia García-Herrera, Mercedes M. Pedrosa, Claudia Arribas, José De J. Berrios, Montaña Cámara, Virginia Fernández-Ruiz, Patricia Morales

**Affiliations:** 1 Departmento Nutrición y Ciencia de los Alimentos, Facultad de Farmacia, Universidad Complutense de Madrid (UCM), Pza. Ramón y Cajal, s/n, 28040 Madrid, Spain; mariaciudad@ucm.es (M.C.-M.); erimavega@gmail.com (E.N.V.); patrigar@pdi.ucm.es (P.G.-H.); mcamara@ucm.es (M.C.); vfernand@ucm.es (V.F.-R.); 2 Departmento Tecnología de Alimentos, INIA-CSIC, Ctra. de La Coruña km 7.5, 28040 Madrid, Spain; mmartin@inia.es (M.M.P.); arribas.claudia@inia.es (C.A.); 3 Western Regional Research Center, Agricultural Research Service, United States Department of Agriculture (USDA-ARS-WRRC), 800 Buchanan Street, Albany, CA 94710-1105, USA; jose.berrios@usda.gov

**Keywords:** pulses, gluten-free, extrusion, dietary fiber, arabinoxylans, oligosaccharides

## Abstract

Extrusion cooking allows the development of value-added products from pulses, such as gluten-free snacks with added functional properties. The main objective of this study was to evaluate the changes induced by the extrusion process on the carbohydrate fraction (total carbohydrates, soluble sugars and oligosaccharides, dietary fiber, and arabinoxylans) of novel flour formulations based on chickpeas and rice enriched with different dietary fiber sources. Moreover, the influence of the addition of fiber-rich ingredients, such as Fibersol^®^ and passion fruit, on the analyzed compounds was also evaluated. Sucrose was the main soluble sugar found in analyzed formulations, and raffinose was the prevalent oligosaccharide, followed by stachyose. The content of total α-galactosides tended to be higher after extrusion cooking. As a consequence of the extrusion treatment, the content of total and soluble dietary fiber was statistically increased in most of the analyzed samples. In general, no significant changes were observed in total arabinoxylan content as a consequence of the extrusion process, while the content of water-soluble arabinoxylans was significantly increased in extruded formulations. It was observed that the content of total available carbohydrates, stachyose, and water-soluble arabinoxylans were significantly influenced by the addition of passion fruit, Fibersol^®^, and both. The incorporation of these ingredients in gluten-free formulations based on chickpeas and rice allows one to obtain suitable functional formulations for the development of innovative, gluten-free, extruded snack-type products, which could be an interesting alternative for people with celiac disease.

## 1. Introduction

Extrusion is a versatile, high-temperature–short-time technology that allows the production of fully cooked, low-moisture, and shelf-stable food products [1,2]. Nowadays, extrusion is one of the most interesting technological processes used by the food industry for the fabrication of a variety of food products since this technology is suitable for the development of value-added products, from pulses and other grains, including gluten-free snacks, with functional properties, high nutritional value, and attractive organoleptic characteristics [3,4,5,6]. This is relevant as snack-type products have currently become a significant part of the diet of many people around the world, particularly children, and these food products represent an excellent vehicle to improve consumer’s health. For this reason, in recent years, demand for healthier snack products made from pulses has substantially increased [7].

Pulses are staple foods in most developing countries, as well as in those countries with a dominant vegetarian diet, as pulses have high protein content with a good amino acid profile, only being deficient in methionine. For this reason, it is recommended to consume pulses together with cereals to improve their amino acid profile [3,8,9,10]. Moreover, the extruded snacks based on cereal–pulse formulations could be a good strategy to develop shelf-stable, ready-to-eat food products with valuable nutritional profiles. These aspects are particularly important in geographic areas where malnutrition is a serious health problem [11]. In addition, extrusion allows the use of different by-products, such as those derived from passion fruit, which improve the nutritional composition as well as the functional properties of the extrudates [12]. Extrusion also allows the incorporation of different dietary fiber sources, such as Fibersol^®^, which is a water-soluble, nonviscous, and highly digestion-resistant maltodextrin that stimulates the production of satiety hormones and enhances satiety in humans [13].

The incorporation of pulses in extruded flour formulations intended for the development of novel, gluten-free, snack-type products is an attractive alternative for improving the nutritional quality of these food products, as pulses are highly valuable foods due to their remarkable nutritional profile. Pulses have a high content of carbohydrates, including dietary fiber, starch, and soluble sugars. It is well known that the intake of dietary fiber is associated with several health benefits, as it can contribute to the normal function of the intestinal tract, and it plays an important role in the prevention of different diseases, such as cancer, diabetes, and cardiovascular diseases [1,14]. 

Pulses’ oligosaccharides are low-molecular-weight carbohydrates formed by 3–10 sugar units. Their main oligosaccharides are the α-galactosides raffinose, stachyose, verbascose, and ajugose, which are compounds that present a prebiotic effect due to the production of short-chain fatty acids (SCFA), as a result of their fermentation by colonic microbiota. SCFA inhibit the growth of some putrefactive and pathogenic bacteria and stimulate the growth of bifidobacteria, which contribute positively to human health. Bifidobacteria present potential protective effects against colorectal cancer and infectious bowel diseases. In addition, SCFA contribute to lowering cholesterol and blood glucose levels [3,4,14].

Pulses also contain arabinoxylans (AX), which are non-cellulosic polysaccharides that are part of the soluble fraction of dietary fiber [5,15]. These compounds are constituted by a linear chain of β-d-(1 → 4)- xylopyranose that is substituted on the hydroxyl groups (–OH) of the 2- and 3-positions by L-arabinofuranosyl residues linked by β-(1 → 4) glycosidic bonds. Moreover, residues of ferulic acid are often found in position 5. According to their physical properties, in particular their solubility, AX could be classified as extractable in water (WEAX) or non-extractable in water (WUAX) [14]. In recent years, AX have been widely studied due to their nutritional and functional properties [10,16]. One of the most important health benefits related to AX are their prebiotic properties, as they are resistant to digestion and are fermentable by gut microbiota. These prebiotic benefits and the antioxidant and immunomodulatory properties of AX are associated with their possible role in the prevention of some types of cancer, including colon cancer, liver cancer, glandular stomach cancer, and neuroblastoma [17]. In addition, AX can selectively stimulate the growth and/or activity of beneficial intestinal bacteria, including *Bifidobacterium* or *Lactobacillus* [14,18]. AX also exhibit their beneficial health effects by regulating glycemic and lipidic metabolism [19,20].

The novelty of this study resides in the new extruded formulation design based in the scientific knowledge that cereals (such as rice) and pulses (such as chickpeas) with optimum ratios of proteins, as used in the study, combined with the use of different amounts of soluble fiber (Fibersol^®^) and a source of insoluble fiber (passion fruit). The main objective of this study was to evaluate the changes induced by the extrusion process on the carbohydrate fraction (total carbohydrates, soluble sugars and oligosaccharides, dietary fiber, and arabinoxylans) of novel formulations based on chickpea–rice flours. Moreover, the influence of the added, fiber-rich ingredients, passion fruit and Fibersol^®^, to obtain suitable functional formulations for the development of innovative, gluten-free, snack-type products, was evaluated. Gluten-free snack products could be a suitable food alternative to commercial gluten-containing snacks for people with celiac disease.

## 2. Results and Discussion

### 2.1. Extrusion Effects on Total Available Carbohydrates in Formulated Flours

The content of total available carbohydrates in the analyzed gluten-free, raw flours based on chickpeas–rice (Table 1) ranged from 77.65 to 93.86 g of glucose per 100 g of sample. These values corresponded to formulations CF#8 (formulated with 71.25% chickpea–rice blend, 12.5% passion fruit, and 10% Fibersol^®^) and CR (formulated with 93.75% chickpea–rice blend, 0% passion fruit, and 0% Fibersol^®^), respectively, while in the corresponding extruded flours (Table 2), the values of total available carbohydrates ranged from 75.55 to 89.77 g of glucose per 100 g of sample for samples EF#3 (83.75% chickpea–rice blend, 5% passion fruit, and 5% Fibersol^®^) and EF#5 (73.75% chickpea–rice blend, 12.5% passion fruit, and 7.5% Fibersol^®^), respectively. In general, no statistically significant (*p* < 0.05) differences were observed in total available carbohydrates between extruded samples formulated with the highest percentages of Fibersol^®^ (7.5% and 10%), while in those samples formulated with the lowest amount of this compound (0% and 5%), a decrease (*p* < 0.05) in total available carbohydrates was observed as a consequence of extrusion. Berrios et al. (2010) observed a decrease in total available carbohydrate content in lentil, chickpea, and dry pea flours after extrusion at 160 °C [1], which agrees with the results of the present study. They also indicated that the reduction in total available carbohydrates in the extruded chickpea flour was significant (*p* < 0.05), probably due to the Maillard condensation reaction, as previously stated by Frias et al. (2011) [21]. On the other hand, Arribas et al. (2019) reported that the effect caused by extrusion treatment depends on the processing conditions, as well as the food matrix analyzed [22]. These authors reported similar values of total carbohydrates as those determined in the present study, of 74.89 to 84.07 g/100 g, in different flours based on rice fortified with carob fruit and beans [22]. However, they reported an increase in total carbohydrates after extrusion at 125 °C. The same tendency was reported by Morales et al. (2015) with different lentil flour formulations, with values of total carbohydrates between 68.94 to 71.63 g/100 g after extrusion at 160 °C [23]. These authors considered that mechanical–structure modification induced by cell rupture and higher cell-wall porosity, increased the specific surface area that facilitated the diffusion of solvent inside the extrudates, improving carbohydrate extraction [23]. The results obtained in the present work and those previously reported by other authors show that extrusion conditions, as well as the characteristics of the food matrix of the material under processing, influence the carbohydrate content of the final products.

### 2.2. Extrusion Effects on Soluble Sugars and Oligosaccharides

The main soluble sugar found in the analyzed formulations was sucrose, and its content ranged from 32.44 to 40.49 mg/g in the raw samples and from 40.41 to 53.94 mg/g in the extruded samples (Table 1). Maltose was only detected in samples CR (1.59 mg/g), CF#7 (0.61 mg/g) and CF#9 (0.63 mg/g), while galactinol was detected in samples CR (1.89 mg/g), CF#1 (6.50 mg/g), EF#6 (3.23 mg/g), CF#7 (3.99 mg/g), and CF#9 (2.98 mg/g). In the raw formulated flours, the content of ciceritol ranged from 4.90 to 16.93 mg/g (samples CF#5 and CR, respectively). However, in the case of extruded formulated flours, the content of ciceritol ranged from 7.62 to 16.91 mg/g (samples EF#4 and CE, respectively). Regarding α-galactosides, raffinose was the main sugar, followed by stachyose. Raffinose content in the raw samples varied from 3.54 to 7.21 mg/g (samples CF#1 and CF#8, respectively); and in the extruded samples, the content of raffinose varied from 4.48 to 8.28 mg/g (samples EF#6 and EF#9, respectively), while stachyose content in raw samples varied from 2.17 to 9.20 mg/g (samples CF#5 and CR, respectively), and in the extruded samples, the content varied from 2.17 to 8.04 mg/g (samples EF#5 and CE, respectively).

The profile of soluble sugars and oligosaccharides of different gluten-free formulations based on pulse flours has been widely studied in the last few years, as the interest of the food industry in gluten-free food products has increased due to the demand of this type of product by consumers concerned with and/or suffering from celiac disease. Berrios et al. (2010) analyzed the sugar content of different pulse flours (lentil, dry pea, or chickpea) formulated with specific food ingredients, such as special starch, dietary fiber, and flavoring agents [1]. These authors reported that sucrose was the major disaccharide in the raw pulse flours. Ciudad-Mulero et al. (2020b) have also confirmed that sucrose was the major disaccharide in lentil formulations enriched with nutritional yeast [2]. In addition, sucrose was also the main soluble sugar found in black bean flours [24]. However, in the case of galactosides, the reports of previous authors have not been as consistent as those regarding with sucrose as some of them reported a higher content of stachyose than raffinose in different raw chickpea flours [1,25,26,27,28].

Arribas et al., 2019 and Ciudad-Mulero et al., (2020b) have previously reported that extrusion-processing conditions and the food matrix composition of the feed influence the soluble sugars’ concentration in the final extrudates. [2,22]. The data of sucrose content, presented in Table 1, show that the content of this sugar was statistically (*p* < 0.05) higher in the extruded flours in comparison with their corresponding unprocessed samples. Ai et al. (2016) also observed an increase in the content of sucrose in flours formulated with Medalist Navy Bean as a consequence of extrusion processing. These same authors studied the effect of eight different extrusion conditions on the chemical composition and functional properties of several flours obtained from four different bean varieties [29]. They reported values of sucrose lower than those found in the present study in both raw and extruded bean flours [29]. Other authors have reported that the concentration of sucrose was not altered after the extrusion processing of wheat–lentil flours [30]. However, a decrease in the content of sucrose was reported by Ciudad-Mulero et al. (2020b) in extruded flours formulated with lentil and nutritional yeast [2] and also in extruded flours of chickpea formulations [1]. In the present study, the effect of extrusion on the content of ciceritol was variable (Table 1). No significant difference (*p* < 0.05) was determined in samples CE, EF#2, or EF#3, whereas the content of this trisaccharide-type sugar-alcohol was significantly increased (*p* < 0.05) after extrusion in formulations EF#1, EF#4, EF#5, EF#7, and EF#9. Moreover, the content of ciceritol was statistically lower (*p* < 0.05) in samples EF#6 and EF#8, compared to their respective raw flours. Similar observations were previously reported by Ciudad-Mulero et al. (2020b), who determined that the content of ciceritol on different lentil-based formulations did not follow a consistent pattern [2]. These results present an interesting case for subsequent studies to elucidate the reason for such inconsistent results.

In this study, as a consequence of the technological treatment, the content of raffinose was statistically higher (*p* < 0.05) in extruded samples CE, EF#1, EF#5, EF#7, and EF#9, compared with their respective raw formulations. Furthermore, the content of stachyose was statistically increased (*p* < 0.05) after extrusion treatment in samples EF#1, EF#2, EF#3, EF#4, and EF#7. However, Berrios et al. (2010) observed that the oligosaccharide content in different pulse flours tended to decrease as a consequence of the relatively high temperature (160 °C), screw speed (500 rpm) and relatively low feed moisture (17%) [1]. In particular, these authors found that the content of stachyose in chickpea formulations decreased from 12.20 to 7.68 mg/g as a consequence of extrusion [1]. Moreover, the results obtained by Ai et al. (2016) showed that extrusion significantly reduced the raffinose content but increased the stachyose content in different bean flours [29]. In samples analyzed in the present study, the content of total α-galactosides tended to be higher after extrusion cooking. This increase was statistically significant (*p* < 0.05) in samples EF#1, EF#4, EF#5, EF#7, and EF#9. This fact is interesting as these compounds have beneficial effects on human health due to their prebiotic properties [31], and it could be because the extrusion-process conditions improve the extractability of α-galactosides in the extrudates [2]. Although there is not a recommended daily intake of galactosides, the consumption of up to 3 g/day of galactosides can provide health effects without provoking flatulence discomfort [32]; then, considering one serving of 40 g, the snacks elaborated can supply from 0.6 g (CE) to 0.32 g (EF#5). Other authors have confirmed that extrusion cooking results in an increase of total α-galactoside content in different formulations based on lentil flours [2,4], but it has been also reported that extrusion did not affect the concentration of raffinose, stachyose, or verbascose in wheat–lentil flours [30]. Indeed, controversy exists about the effect of extrusion on α-galactosides [29], as it could be dependent on the variety of the pulse and the extrusion conditions.

### 2.3. Extrusion Effects on Dietary Fiber (Total, Soluble and Insoluble Dietary Fiber)

Dietary fiber is constituted by the remnants of the edible part of plants and analogous carbohydrates which resist digestion and absorption in the human small intestine and are completely or partially fermented in the human large intestine [14,33,34]. 

In the present study, we have analyzed the content of total (TDF), soluble (SDF), and insoluble dietary fiber (IDF) in different gluten-free formulations (raw and extruded) based on rice and chickpea flours, and the results are shown in Table 2. In raw samples, TDF content ranged from 8.65 g/100 g in sample CF#9 (formulated with 78.75% chickpea–rice blend, 5% passion fruit, and 10% Fibersol^®^) to 16.74 g/100 g in sample CF#8 (formulated with 71.25% chickpea–rice blend, 12.5% passion fruit, and 10% Fibersol^®^). All analyzed samples contained at least 6 g of fiber per 100 g, and for this reason, they could be considered to be food products high in fiber, according to Regulation (EC) No 1924/2006 [35]. This fact indicates that the analyzed formulations have potential functional properties, and their incorporation into the development of new, functional food products could be very interesting from a nutritional point of view. Similar values of TDF have been previously reported by other authors in different multigrain mixes prepared from different cereals, pulses, millets, and nuts (12.4 to 16.5 g of TDF per 100 g) [36], in lentil flour formulations fortified with fiber-rich ingredients (9.32 to 12.42 g of TDF per 100 g) [23], in lentil formulations enriched with nutritional yeast (13.11 to 18.39 g of TDF per 100 g) [5], in expanded snacks based on corn–common bean flour mixtures (9.57 to 14.18 g of TDF per 100 g) [7], and in gluten-free snacks based on a plantain, chickpea, and maize blend (13.71 to 18.20 g of TDF per 100 g) [37].

Regarding the different dietary fiber fractions, IDF was the prevalent fraction in all analyzed formulations (4.46 to 11.47 g/100 g of raw flour, in samples CF#9 and CF#1, respectively), while SDF was found in lower amounts (1.56 to 5.82 g/100 g of raw flour, in samples CF#4 and CF#8, respectively). This is a common pattern in the dietary fiber composition of pulses [1,5], and several studies have shown values of IDF and SDF in pulse formulations which are in accordance with the values found in the present study. In this manner, Itagi & Singh (2012) studied different formulations based on cereals and pulses (rice, wheat, ragi, chickpeas, whole green gram, puffed Bengal gram, defatted soya powder, etc.), among other ingredients, and reported values of 9.7 to 14.0 g of IDF/100 g and 2.4 to 2.7 g of SDF/100 g [36]. Moreover, in flours based on lentils, amounts of IDF between 8.93 to 14.3 g/100 g and values for SDF between 2.58 to 4.62 g/100 g [5] have been found. In addition, similar concentrations have been observed in snacks based on cereal–pulse flours (from corn and common beans) (8.90 to 9.53 g of IDF per 100 g and 0.66 to 2.15 g of SDF per 100 g) [7].

As a consequence of extrusion cooking, the content of TDF was statistically increased (*p* < 0.05) in most of the analyzed samples, achieving values between 8.72 g/100 (sample EF#4) and 18.29 g/100 g (sample EF#1). This increment was mainly due to the higher content (*p* < 0.05) of SDF found in the extruded samples (except for flours EF#7, EF#8, and EF#9, formulated with 10% Fibersol^®^). This represented a 209% increase from the case of the control sample (CE). After extrusion, SDF content varied between 3.03 to 6.54 g/100 g, in samples EF#4 and EF#5, respectively. In the case of IDF (5.69 to 14.16 g/100 g, in extruded samples EF#4 and EF#1, respectively), a steady trend after extrusion treatment was not observed. Similar observations have been previously reported by other authors. For example, Ciudad-Mulero et al. (2018) analyzed different lentil flour formulations and reported that extrusion caused an increase in TDF and SDF content, while the effect of this technology on the IDF content did not follow a consistent trend [5]. Extrusion processing significantly influenced the dietary fiber profile of lupin kernel fiber, as this treatment increased the proportion of SDF [38]. Furthermore, the same effect has been observed in the case of lupin seed coat [39]. The increment in TDF and SDF content could be due to the releasing of gums and mucilages as a consequence of the extrusion conditions (temperature, moisture content, high pressure, screw speed, and cutting) [40]. In addition, the increase in the content of SDF in the extrudates could be attributed to the higher amounts of oligosaccharides, which resulted from the breakdown of the polysaccharides’ glycosidic bonds [6]. Moreover, gelatinization can occur during the extrusion process, and this physicochemical modification could also explain the increase in the SDF fraction [5].

### 2.4. Extrusion Effects on Arabinoxylans

In recent years, arabinoxylans (AX), which are one of the most noteworthy compounds within the soluble dietary fiber constituents, have aroused great interest among the scientific community due to their potentially protective role against several diseases, such as diabetes, cardiovascular diseases, and certain types of cancer [14].

In the present study, we quantified the content of total arabinoxylans and water-soluble arabinoxylans, and the obtained results are shown in Table 2. The concentration of total arabinoxylans in raw analyzed gluten-free formulations ranged from 6.76 (samples CF#6 and CF#9) to 9.73 g/100 g (sample CF#3). On the other hand, in the case of extruded samples, these values were between 7.88 to 9.77 g/100 g (formulations EF#6 and EF#5, respectively). There are a very few published studies regarding AX content in pulses. Ciudad-Mulero et al. (2018) reported slight decreases in total arabinoxylans concentrations in lentil formulations enriched with nutritional yeast (3.67 to 7.18 g/100 g of raw flour, and 5.79 to 7.68/100 g of extruded flour) [5]. This difference could be related to the presence of rice in the analyzed formulations, as arabinoxylans are the main noncellulosic polysaccharides present in cereals [14]. In another study, Dodevska et al. (2013) reported values of total arabinoxylans of 2.21, 1.03, 1.07, and 0.97 g/100 g in cooked kidney beans, lentils, peas, and string beans, respectively [41]. 

Regarding water-soluble arabinoxylans, the content of these compounds in raw analyzed flours varied between 0.80 to 3.00 g/100 g, values in accordance with those obtained in different lentil flours (1.07 to 1.44 g/100 g) [5].

No significant changes (*p* < 0.05) were observed in total arabinoxylan content as a consequence of the extrusion treatment, with the exception of samples CE, EF#6, and EF#9, whose content was statistically higher (*p* < 0.05), compared to their respective raw counterparts. Moreover, the content of water-soluble arabinoxylans was significantly (*p* < 0.05) increased as a consequence of extrusion cooking, with these values ranging from 1.78 (sample EF#7) to 4.24 (sample EF#2) g/100 g in extruded formulations. This extrusion effect has been previously reported by other authors in different food matrices, such as lentil flour [5], wheat bran [42], or barley flour [43]. Moreover, it has been observed that the extraction of arabinoxylans was significantly increased with an increase in screw speed, and it was accompanied by a significant decrease in the molecular weight of arabinoxylans from extruded rice bran [44].

### 2.5. Effect of Ingredients on Chemical Composition of Analyzed Formulations

As shown in Table 3, significant effects of passion fruit and Fibersol^®^ addition were found on the chemical composition of analyzed gluten-free formulations (*p* ≤ 0.05). No significant effect of the incorporation of passion fruit and Fibersol^®^ was observed on the content of galactinol, raffinose, or total dietary fiber. In addition, no significant interactions were found for passion fruit addition with Fibersol^®^ incorporation in the case of total α-galactoside and total arabinoxylan content, while these compounds were significantly affected by the individual enrichment with passion fruit and Fibersol^®^. Furthermore, the presence of passion fruit in the analyzed gluten-free formulations significantly impacted the content of insoluble dietary fiber (*p* = 0.0016), while the presence of Fibersol^®^ significantly affected the content of sucrose (*p* = 0.0178), ciceritol (*p* = 0.0000), and soluble dietary fiber (*p* = 0.050). Finally, it was observed that the content of total available carbohydrates, stachyose, and water-soluble arabinoxylans was significantly influenced (*p* ≤ 0.05) by the addition of passion fruit, Fibersol^®^, and both.

The use of a combination of a soluble fiber (Fibersol^®^) and a source of insoluble fiber (passion fruit) presents a very interesting study regarding their contribution as carbohydrate fractions resulting as a consequence of extrusion processing. 

## 3. Materials and Methods

### 3.1. Formulated Flours Composition

Different formulations were developed for extrusion processing by combining and properly mixing the chickpea–rice (30:70) flours with different percentages of Fibersol^®^ (Chicago, IL, USA) and passion-fruit-skin flour, which henceforth will be referred to as passion fruit (Table 4). All formulated flours (from raw and extruded material) were reduced to uniform powders using a Cyclone Mill (Udy Corp., Fort Collins, CO, USA) fitted with a 0.5-mm screen, and then stored in air-tight glass jars at room temperature until analyzed.

### 3.2. Extrusion Process Conditions

Extrusion cooking was carried out using a Clextral EVOL HT32-H twin-screw extruder (Clextral, Inc., Tampa, FL, USA) equipped with six barrel sections (each 128 mm in length) with co-rotating and closely intermeshing screws. In order to set the number of variables for moisture content of the mixtures (screw speed and die temperature), the control formulation was previously pre-run under different extrusion processing conditions (data not shown). Then, the determined optimum conditions for total moisture, screw speed, and temperature were used to run all the formulations reported in the study. The die and the last barrel section maintained a temperature of 140 ± 1 °C. The diameter of the screw (D) was 32 mm. and total screw length (L) was 768 mm, resulting in an L/D ratio of 24. Screws were driven by a 74.8 kW variable-speed drive, Model ACS600 (ABB Automation, Inc., New Berlin, WI, USA). The screw speed was maintained constant at 500 rpm. A combination of feeding, transporting, compression, and kneading elements was utilized to procure a moderate-shear screw configuration (patent pending) [45].

The mixture was metered into the feed port by a twin-screw, loss-in-weight gravimetric feeder, Model LWFD5-20 (K-Tron Corp., Pitman, NJ, USA) at a rate of 20 kg/h (wwb). Water was provided to the extruder by a triplex variable stroke piston pump with 12 mm plungers, Type VE-P33 (Bran and Luebbe, Wheeling, IL, USA) to provide a final feed moisture content of 17 percent. The gluten-free formulations based on chickpea–rice enriched with passion fruit and Fibersol^®^ were extruded through two circular dies, each with a 3.5 mm-diameter opening. Samples were collected approximately 10 min after the operation conditions of torque and pressure were at a steady state.

### 3.3. Chemical Analysis

#### 3.3.1. Analysis of Total Available Carbohydrates

Carbohydrates were calculated using the anthrone method, following the procedure described by Berrios et al. (2010) [1]. In this method, 0.4 g of the samples was added to perchloric acid and left overnight to digest. After filtration, anthrone reagent (9,10-dihydro-9-oxoanthracene) was added to test tubes containing the samples, which were first boiled and then cooled. The detection of total available carbohydrates was carried out on a spectrophotometer (EZ210 PerkinElmer, Waltham, MA, USA) at a wavelength of 630 nm. Results of their analysis were expressed as grams of glucose per 100 g of fresh weight.

#### 3.3.2. Analysis of Soluble Sugars and Oligosaccharides

The concentration of soluble sugars and oligosaccharides in the raw and extruded flours was determined by high-performance liquid chromatography (HPLC), using a procedure described by Muzquiz et al. (1992) [46] and modified by Pedrosa et al. (2012) [27]. The extraction was carried out using a 50% *v*/*v* aqueous ethanol, and the residue obtained, after evaporation and dryness, was redissolved in 1 mL of double-deionized water and centrifuged for 10 min at 10,000 rpm. After that, the obtained samples were filtered through a 0.45 µm Millipore membrane, and 20 µL of each extract was injected into an HPLC system (Beckman System Gold Instrument, Los Angeles, CA, USA) equipped with a Spherisorb-5-NH2 column (250 × 4.6 mm i.d., Waters, Milford, MA, USA), equilibrated with acetonitrile/water 60:40 (*v*/*v*), and a refractive index detector. The flow rate was 1 mL/min and the samples were analyzed in duplicate. Calibration curves for all standard sugar solutions were prepared. Individual sugars were quantified by comparison with external standards of pure sucrose, maltose, raffinose, stachyose. and verbascose (Sigma, St. Louis, MO, USA); ciceritol and galactinol were purified and kindly supplied by Dr. A. I. Piotrowicz-Cieslak (Olsztyn-Kortowo, Poland).

#### 3.3.3. Analysis of Dietary Fiber (Total, Soluble and Insoluble Dietary Fiber)

Total, soluble, and insoluble dietary fiber were determined according to AOAC enzymatic–gravimetric methods 993.19 and 991.42 [1,47]. An amount of 0.3 g of each sample was placed in a 250 mL Erlenmeyer flask, and 50 mL of phosphate buffer (pH = 6.0 ± 0.2) solution and 0.1 mL of α-amylase was added to the samples. The samples were incubated under shaking at ∼100 °C for 15 min. After that, the pH of the solution was adjusted to pH = 7.5 ± 0.2 with 10 mL of 0.275 N NaOH, and 5 mg of protease was added. After re-incubation at 60 °C under shaking for 30 min, the pH was re-adjusted to pH = 4–4.6 with 10 mL of 0.325 N HCl and 0.1 mL of amyloglucosidase. The mixture was filtered (insoluble fiber), and the filtrate was collected in a 500 mL Erlenmeyer flask and the suspension precipitated by the addition of 400 mL ethanol (soluble fiber). Samples were run in duplicate, and protein and ash were subtracted for the calculation of total dietary fiber (TDF) content. The values were expressed as g per 100 g flour.

#### 3.3.4. Analysis of Arabinoxylans

Total arabinoxylans (TOAX) and water-soluble arabinoxylans (WEAX) were quantified using a colorimetric method. An amount of 100 mg of each sample was placed in a conical polypropylene tube, and 25 mL of distilled water was added. After shaking for 30 min, 0.5 mL of the sample suspension was quickly removed and pipetted into a stoppered reaction tube in order to determinate the TOAX content. The original sample suspension was centrifuged for 10 min at 2500 rpm (Universal 16 R, Genesys Instrumentation, SL, Madrid, Spain). After centrifugation, 0.5 mL of the supernatant was removed and pipetted into an amber reaction tube for determination of WEAX content. Once the sample aliquots were collected, 1.5 mL of distilled water was added to bring the final volume to 2 mL. The calibration curve was prepared using a stock solution of 10 mg of d-(+)-xylose in 100 mL of distilled water. Then, triplicate standard samples were prepared using different concentrations of xylose (0.005–1 mg mL^−1^). The absorbance was measured at 448 and 508 nm for determination of the arabinoxylans. In order to remove the influence of hexoses (mainly glucose), the absorbance reading at 508 nm was subtracted from the sample readings at 448 nm. The referred wavelengths were selected after performing a full scanning to check the maximum absorbance of the sugars. The values were expressed as g per 100 g flour. 

### 3.4. Statistical Analysis

Mean ± standard deviations (SD) of the sample data were determined and statistically analyzed by Analysis of variance (ANOVA). In addition, two-way interactions were included in the ANOVA in order to examine the impact of the ingredients added to the formulations for enrichment (passion fruit and Fibersol^®^). Duncan’s Multiple Comparison Test was applied to identify the differences among the sets of means. All statistical analyses were performed using Statgraphics Plus 5.1 software (Statgraphics Technologies, Inc., The Plains, VA, United States). The statistical significance level was set at *p* < 0.05.

## 4. Conclusions

In recent years, demand for healthier products in the snack market has increased substantially, with most of those made from pulse flours. In this sense, the food industry is required to innovate by using new processing technologies, as well as novel ingredients, to develop innovative products that meet the consumers’ demand. The results of this study showed that the extrusion process is an interesting technology which allows one to obtain suitable gluten-free formulations with added functional properties, as extruded flours based on rice and chickpeas were higher in dietary fiber, water soluble arabinoxylans, and oligosaccharides. Moreover, the chemical composition of analyzed gluten-free formulations has been influenced by the addition of fiber-rich ingredients, such as Fibersol^®^ and passion fruit. Therefore, the incorporation of these ingredients in extruded gluten-free formulations based on rice and chickpeas could be an interesting alternative so as to increase the range of snack-type products marketed for people with celiac disease. The study of the morphology of the extrudates, as well as the use of other processing temperatures, would be additional interesting aspects of a future study to relate morphology and effect of temperature on resulted extrudates.

## Figures and Tables

**Table 1:** The content of soluble sugars (mg/g, fresh weight), α-galactosides (mg/g, fresh weight), and total available carbohydrates (g/100 g, fresh weight) in fortified rice–chickpea flours (raw and extruded) (mean ± SD).

Sample	Sucrose	Maltose	Galactinol	Raffinose	Ciceritol	Stachyose	Total α-Galactosides	Total Available Carbohydrates
**CR**	40.49 ± 1.86 ^c,A^	1.59 ± 0.26	1.89 ± 0.02	5.51 ± 0.12 ^c,A^	16.93 ± 0.19 ^g,A^	9.20 ± 0.42 ^e,B^	14.71 ± 0.54 ^f,A^	93.86 ± 5.23 ^e,B^
**CE**	49.83 ± 0.79 ^e,B^	nd	nd	7.25 ± 0.10 ^de,B^	16.91 ± 0.85 ^f,A^	8.04 ± 0.71 ^f,A^	15.29 ± 0.63 ^f,A^	82.22 ± 7.4 ^bcd,A^
**CF#1**	36.11 ± 1.61 ^b,A^	nd	6.50 ± 0.16	3.54 ± 0.14 ^a,A^	9.19 ± 0.30 ^d,A^	5.34 ± 0.04 ^d,A^	8.88 ± 0.18 ^b,A^	93.85 ± 5.27 ^e,B^
**EF#1**	44.14 ± 0.36 ^c,B^	nd	nd	7.07 ± 0.36 ^de,B^	16.10 ± 0.98 ^f,B^	7.25 ± 0.44 ^e,B^	14.31 ± 0.13 ^e,B^	84.11 ± 2.76 ^cd,A^
**CF#2**	40.04 ± 1.44 ^c,A^	nd	nd	6.82 ± 0.19 ^e,B^	13.69 ± 0.56 ^f,A^	5.73 ± 0.46 ^d,A^	12.55 ± 0.60 ^e,A^	92.36 ± 3.92 ^de,B^
**EF#2**	43.34 ± 0.21 ^b,B^	nd	nd	5.98 ± 0.55 ^bc,A^	13.12 ± 0.47 ^e,A^	6.73 ± 0.37 ^d,B^	12.71 ± 0.89 ^cd,A^	84.54 ± 6.32 ^cd,A^
**CF#3**	37.96 ± 1.13 ^bc,A^	nd	nd	6.96 ± 0.54 ^e,A^	13.23 ± 0.35 ^f,A^	5.55 ± 0.19 ^d,A^	12.51 ± 0.52 ^e,A^	90.05 ± 4.73 ^cde,B^
**EF#3**	43.66 ± 0.38 ^bc,B^	nd	nd	7.15 ± 0.65 ^de,A^	12.44 ± 0.74 ^e,A^	6.33 ± 0.18 ^cd,B^	13.47 ± 0.69 ^d,A^	75.55 ± 4.75 ^a,A^
**CF#4**	32.44 ± 0.36 ^a,A^	nd	nd	6.06 ± 0.72 ^d,A^	6.35 ± 0.23 ^b,A^	2.49 ± 0.39 ^a,A^	9.16 ± 0.50 ^b,A^	85.01 ± 4.87 ^b,B^
**EF#4**	40.75 ± 0.42 ^a,B^	nd	nd	6.61 ± 0.62 ^cd,A^	7.62 ± 0.53 ^a,B^	3.40 ± 0.19 ^b,B^	10.17 ± 0.64 ^b,B^	80.26 ± 2.90 ^abc,A^
**CF#5**	35.65 ± 0.52 ^b,A^	nd	nd	5.23 ± 0.14 ^c,A^	4.90 ± 0.22 ^a,A^	2.17 ± 0.31 ^a,A^	7.40 ± 0.25 ^a,A^	88.67 ± 3.18 ^bcd,A^
**EF#5**	40.41 ± 0.36 ^a,B^	nd	nd	5.89 ± 0.52 ^b,B^	8.60 ± 0.58 ^b,B^	2.17 ± 0.18 ^a,A^	8.05 ± 0.46 ^a,B^	89.77 ± 3.47 ^e,A^
**CF#6**	40.09 ± 3.93 ^c,A^	nd	nd	7.13 ± 0.61 ^e,B^	10.92 ± 0.35 ^e,B^	4.09 ± 0.27 ^b,B^	11.21 ± 0.75 ^d,B^	85.67 ± 4.19 ^bc,A^
**EF#6**	48.20 ± 0.45 ^d,B^	nd	3.23 ± 0.30	4.48 ± 0.25 ^a,A^	7.70 ± 0.09 ^a,A^	3.67 ± 0.17 ^b,A^	8.16 ± 0.42 ^a,A^	86.51 ± 4.62 ^de,A^
**CF#7**	36.17 ± 0.35 ^b,A^	0.61 ± 0.17	3.99 ± 0.09	4.47 ± 0.22 ^b,A^	6.46 ± 0.26 ^b,A^	4.86 ± 0.23 ^c,A^	9.33 ± 0.41 ^b,A^	78.60 ± 2.98 ^a,A^
**EF#7**	50.54 ± 0.28 ^f,B^	nd	nd	6.90 ± 0.37 ^d,B^	11.55 ± 0.52 ^d,B^	6.17 ± 0.34 ^c,B^	13.07 ± 0.66 ^d,B^	78.71 ± 3.29 ^bc,A^
**CF#8**	32.62 ± 0.34 ^a,A^	nd	nd	7.21 ± 0.37 ^e,A^	11.34 ± 0.56 ^e,B^	4.42 ± 0.33 ^b,B^	11.63 ± 0.51 ^d,B^	77.65 ± 3.93 ^a,A^
**EF#8**	50.93 ± 0.43 ^f,B^	nd	nd	7.69 ± 0.53 ^ef,A^	10.39 ± 0.42 ^c,A^	2.55 ± 0.21 ^a,A^	10.33 ± 0.49 ^b,A^	77.66 ± 4.34 ^bc,A^
**CF#9**	35.56 ± 1.04 ^b,A^	0.63 ± 0.20	2.98 ± 0.24	5.62 ± 0.21 ^cd,A^	7.39 ± 0.22 ^c,A^	4.38 ± 0.27 ^b,B^	10.00 ± 0.38 ^c,A^	78.08 ± 2.31 ^a,A^
**EF#9**	53.94 ± 0.46 ^g,B^	nd	nd	8.28 ± 0.35 ^f,B^	13.19 ± 0.61 ^e,B^	3.68 ± 0.25 ^b,A^	11.96 ± 0.38 ^c,B^	78.14 ± 2.55 ^bc,A^

In each column, different letters mean statistically significant differences (*p* < 0.05) compared by Duncan’s test; lowercase superscript letter means differences between all samples of the same treatment (raw or extruded), whereas uppercase superscript letter means difference due to extrusion treatment for the same formulation. nd: non detected.

**Table 2:** Dietary fiber (insoluble, soluble, and total) and arabinoxylan (total and water-soluble) content in fortified rice–chickpea flours (raw and extruded) (g/100 g, fresh weight) (mean ± SD).

Sample	Insoluble Dietary Fiber	Soluble Dietary Fiber	Total Dietary Fiber	Water Soluble Arabinoxylans	Total Arabinoxylans
**CR**	8.50 ± 0.18 ^d,A^	1.57 ± 0.09 ^a,A^	10.07 ± 0.18 ^b,A^	1.85 ± 0.18 ^c,A^	8.19 ± 0.81 ^b,A^
**CE**	8.13 ± 0.72 ^bc,A^	4.85 ± 0.44 ^bc,B^	12.98 ± 0.72 ^c,B^	2.50 ± 0.21 ^bc,B^	9.62 ± 0.80 ^cd.B^
**CF#1**	11.74 ± 1.05 ^f,A^	2.82 ± 0.17 ^b,A^	14.56 ± 1.05 ^d,A^	1.73 ± 0.14 ^c,A^	9.21 ± 0.37 ^cde,A^
**EF#1**	14.16 ± 1.20 ^f,B^	4.12 ± 0.19 ^b,B^	18.29 ± 1.20 ^f,B^	3.76 ± 0.28 ^f,B^	9.42 ± 0.31 ^cd,A^
**CF#2**	10.18 ± 0.92 ^e,B^	2.47 ± 0.23 ^b,A^	12.65 ± 0.92 ^c,A^	0.80 ± 0.04 ^a,A^	8.85 ± 0.51 ^bcd,A^
**EF#2**	7.62 ± 0.33 ^b,A^	5.25 ± 0.14 ^c,B^	12.87 ± 0.33 ^c,A^	4.24 ± 0.38 ^g,B^	9.24 ± 0.32 ^cd,A^
**CF#3**	7.45 ± 0.74 ^c,A^	2.78 ± 0.27 ^b,A^	10.22 ± 0.33 ^b,A^	2.64 ± 0.10 ^d,A^	9.73 ± 0.67 ^e,A^
**EF#3**	10.23 ± 0.49 ^e,B^	6.50 ± 0.14 ^c,B^	16.73 ± 0.49 ^e,B^	3.28 ± 0.30 ^e,B^	9.69 ± 0.81 ^cd,A^
**CF#4**	8.57 ± 0.80 ^d,B^	1.56 ± 0.09 ^a,A^	10.13 ± 0.80 ^b,B^	1.30 ± 0.14 ^b,A^	9.49 ± 0.93 ^de,A^
**EF#4**	5.69 ± 0.52 ^a,A^	3.03 ± 0.30 ^a,B^	8.72 ± 0.60 ^a,A^	2.27 ± 0.10 ^b,B^	8.49 ± 0.81 ^ab,A^
**CF#5**	6.08 ± 0.00 ^b,A^	4.81 ± 0.11 ^d,A^	10.88 ± 0.42 ^b,A^	1.45 ± 0.16 ^b,A^	9.16 ± 0.71 ^cde,A^
**EF#5**	9.58 ± 0.55 ^de,B^	6.54 ± 0.57 ^d,B^	16.13 ± 0.55 ^e,B^	3.27 ± 0.30 ^e,B^	9.77 ± 0.06 ^d,A^
**CF#6**	5.32 ± 0.17 ^ab,A^	3.66 ± 0.17 ^c,A^	9.10 ± 0.17 ^a,A^	3.00 ± 0.12 ^f,A^	6.76 ± 0.24 ^a,A^
**EF#6**	6.10 ± 0.88 ^a,A^	4.77 ± 0.41 ^bc,B^	10.88 ± 0.88 ^b,B^	4.15 ± 0.28 ^g,B^	7.88 ± 0.58 ^a,B^
**CF#7**	8.63 ± 0.62 ^d,A^	5.23 ± 0.63 ^cd,A^	13.86 ± 0.62 ^d,B^	1.75 ± 0.20 ^c,A^	8.68 ± 0.75 ^bc,A^
**EF#7**	8.98 ± 0.73 ^cd,A^	4.21 ± 0.23 ^b,A^	12.85 ± 0.27 ^c,A^	1.78 ± 0.17 ^a,A^	8.96 ± 0.45 ^bc,A^
**CF#8**	10.92 ± 0.86 ^ef,B^	5.82 ± 0.25 ^e,B^	16.74 ± 0.86 ^e,B^	2.53 ± 0.19 ^d,A^	8.12 ± 0.39 ^b,A^
**EF#8**	9.36 ± 0.27 ^de,A^	4.84 ± 0.46 ^bc,A^	14.20 ± 0.27 ^d,A^	2.82 ± 0.29 ^cd,A^	7.94 ± 0.68 ^a,A^
**CF#9**	4.46 ± 0.28 ^a,A^	4.19 ± 0.35 ^cd,A^	8.65 ± 0.28 ^a,A^	2.63 ± 0.27 ^d,A^	6.76 ± 0.40 ^a,A^
**EF#9**	9.04 ± 0.68 ^cd,B^	4.65 ± 0.45 ^bc,A^	13.69 ± 0.02 ^cd,B^	3.15 ± 0.16 ^de,B^	9.41 ± 0.81 ^cd,B^

In each column, different letters mean statistically significant differences (*p* < 0.05) compared by Duncan’s test; lowercase superscript letters mean differences between all samples of the same treatment (raw or extruded), whereas uppercase superscript letters mean differences due to extrusion treatment for the same formulation.

**Table 3:** Summary p-values of ANOVA main effects and double interactions between Fibersol^®^ content and passion fruit content on carbohydrate composition of fortified rice–chickpea flours.

	Fibersol^®^ Content	Passion Fruit Content	Fibersol^®^ Content * Passion Fruit Content
Available carbohydrates	**0.0014**	**0.0002**	**0.0016**
Sucrose	**0.0178**	0.6064	0.7371
Galactinol	0.8985	0.2421	0.4832
Raffinose	0.3039	0.1200	0.6843
Ciceritol	**0.0000**	0.2285	0.7737
Stachyose	**0.0000**	**0.0455**	**0.0250**
Total α-galactosides	**0.0000**	**0.0196**	0.0556
Insoluble dietary fiber	0.0722	**0.0016**	0.2289
Soluble dietary fiber	**0.0050**	0.4124	0.8049
Total dietary fiber	0.9828	0.0654	0.3841
Water soluble arabinoxylans	**0.0001**	**0.0096**	**0.0000**
Total arabinoxylans	**0.0003**	**0.0174**	0.7390

Emboldened numbers indicate significant effects at *p* ≤ 0.05. Fibersol® Content * Passion Fruit Content: means interaction between two factors (Fibersol® Content and Passion Fruit Content).

**Table 4:** Chickpea–rice flour formulations analyzed.

	Sample	Characteristics		
		% Mixture CP:R	% Passion Fruit	% Fibersol^®^
CR	Control raw flour	93.75	0	0
CE	Control extruded flour			
CF#1	Control formulation 1	68.75	20	5
EF#1	Extruded formulation 1			
CF#2	Control formulation 2	76.25	12.5	5
EF#2	Extruded formulation 2			
CF#3	Control formulation 3	83.75	5	5
EF#3	Extruded formulation 3			
CF#4	Control formulation 4	66.25	20	7.5
EF#4	Extruded formulation 4			
CF#5	Control formulation 5	73.75	12.5	7.5
EF#5	Extruded formulation 5			
CF#6	Control formulation 6	81.25	5	7.5
EF#6	Extruded formulation 6			
CF#7	Control formulation 7	63.75	20	10
EF#7	Extruded formulation 7			
CF#8	Control formulation 8	71.25	12.5	10
EF#8	Extruded formulation 8			
CF#9	Control formulation 9	78.75	5	10
EF#9	Extruded formulation 9			

CP:R = mixture of 30% chickpea with 70% rice flours.

## Data Availability

Data sharing is not applicable to this article.

## Sample Availability

The samples analyzed are available from the authors.
